# MULTIPLE MEASURES OF FITNESS ASSOCIATION WITH MUSCLE MRNA SPLICING VARIANTS IN THE GESTALT STUDY

**DOI:** 10.1093/geroni/igad104.0586

**Published:** 2023-12-21

**Authors:** Stefano Donega, Nirad Banskota, Marta Gonzalez-Freire, Ann Zenobia-Moore, Ceereena Ubaida-Mohien, Ken Fishbein, Supriyo De, Luigi Ferrucci

**Affiliations:** National Institute on Aging, National Institutes of Health, Baltimore, Maryland, United States; National Institute on Aging, National Institutes of Health, Baltimore, Maryland, United States; Health Research Institute of the Balearic Islands (IdISBA), Palma, Islas Baleares, Spain; National Institute on Aging, National Institutes of Health, Baltimore, Maryland, United States; National Institute on Aging, National Institutes of Health, Baltimore, Maryland, United States; National Institute on Aging, National Institutes of Health, Baltimore, Maryland, United States; National Institute on Aging, National Institutes of Health, Baltimore, Maryland, United States; National Institute on Aging, National Institutes of Health, Baltimore, Maryland, United States

## Abstract

Regular physical activity has been associated with higher skeletal muscle mass and strength as well as better mitochondrial function. Previous studies in master octogenarian athletes found a higher content of mitochondrial- and reduced spliceosome proteins compared to non-athlete age/sex-matched controls. These findings suggest that higher mitochondrial function is associated with down-regulation of the spliceosome. In this study we aimed to detect mitochondrial respiration associations with expression of specific genes and differentially spliced isoforms. We performed RNA-seq from vastus lateralis skeletal muscle biopsies in 82 male/female participants in the GESTALT study (Genetic and Epigenetic Signatures of Translational Aging Laboratory Testing). We correlated gene expression, transcript usage and splicing events with I) self-reported level of physical activity in daily life, II) peak oxygen consumption assessed during an incremental treadmill stress testing, III) muscle oxidative capacity assessed by p31 MR Spectroscopy and IV) mitochondrial-respirometry assessed on permeabilized muscle fibers, using regression models adjusted for age, gender, and batch. Except for IV), all three mitochondrial measures positively correlated with each other, and were downregulated along the Aging axis. We detected 423, 219, 178 and 226 differentially expressed genes (p-value<0.01), respectively for the 4 mitochondrial fitness measures. Gene-set-enrichment analysis (GSEA) detected up-top TCA cycle and mitochondrial respiration kPCr and mito-respirometry detected the highest number of AS events (448 and 412). Dissimilarities among transcriptomic profile associated to these four bioenergetic outcomes provide insight into the biological mechanisms that connect them to mitochondrial function and suggest that mitochondrial function is not the only correlate of movement behaviors.

